# Normal microRNA Maturation and Germ-Line Stem Cell Maintenance Requires Loquacious, a Double-Stranded RNA-Binding Domain Protein

**DOI:** 10.1371/journal.pbio.0030236

**Published:** 2005-05-24

**Authors:** Klaus Förstemann, Yukihide Tomari, Tingting Du, Vasily V Vagin, Ahmet M Denli, Diana P Bratu, Carla Klattenhoff, William E Theurkauf, Phillip D Zamore

**Affiliations:** **1** Department of Biochemistry and Molecular Pharmacology, University of Massachusetts Medical School, Worcester, Massachusetts, United States of America,; **2** Institute of Molecular Genetics of RAS, Moscow, Russia,; **3** Watson School of Biological Sciences, Cold Spring Harbor Laboratory, Cold Spring Harbor, New York, United States of America,; **4** Program in Molecular Medicine, University of Massachusetts Medical School, Worcester, Massachusetts, United States of America,; Oregon State UniversityUnited States of America

## Abstract

microRNAs (miRNAs) are single-stranded, 21- to 23-nucleotide cellular RNAs that control the expression of cognate target genes. Primary miRNA (pri-miRNA) transcripts are transformed to mature miRNA by the successive actions of two RNase III endonucleases. Drosha converts pri-miRNA transcripts to precursor miRNA (pre-miRNA); Dicer, in turn, converts pre-miRNA to mature miRNA. Here, we show that normal processing of *Drosophila* pre-miRNAs by Dicer-1 requires the double-stranded RNA-binding domain (dsRBD) protein Loquacious (Loqs), a homolog of human TRBP, a protein first identified as binding the HIV *trans*-activator RNA (TAR). Efficient miRNA-directed silencing of a reporter transgene, complete repression of *white* by a dsRNA trigger, and silencing of the endogenous *Stellate* locus by *Suppressor of Stellate,* all require Loqs. In *loqs* ^f00791^ mutant ovaries, germ-line stem cells are not appropriately maintained. Loqs associates with Dcr-1, the *Drosophila* RNase III enzyme that processes pre-miRNA into mature miRNA. Thus, every known *Drosophila* RNase-III endonuclease is paired with a dsRBD protein that facilitates its function in small RNA biogenesis.

## Abstract 2

This and an accompanying paper by Saito et al. identify Loquacious, which encodes a double-stranded RNA binding domain protein, and partners with Dicer-1 in the processing of microRNAs.

## Introduction

MicroRNAs (miRNAs) are 21- to 23-nucleotide single-stranded RNAs that are encoded in the chromosomal DNA and repress cognate mRNA targets [1,2]. They are transcribed as long, hairpin-containing precursors [3] by RNA polymerase II [4–8] and processed in the nucleus by the multidomain RNase III endonuclease Drosha [9]. Drosha is assisted by its double-stranded RNA-binding domain (dsRBD) protein partner, known as Pasha in *Drosophila* *melanogaster* [10] and DGCR8 in humans [11–13]. Exportin-5 (Ranbp21 in *Drosophila*) binds the resulting precursor miRNA (pre-miRNA)—likely recognizing the approximately two-nucleotide 3′ overhanging ends characteristic of these approximately 70-nucleotide hairpin structures—and transports them to the cytoplasm via the Ran-GDP–Ran-GTP transport system [14–16]. In the cytoplasm, a second RNase III endonuclease, Dicer, converts pre-miRNA into mature miRNA [17–20].

In *Drosophila,* two Dicer paralogs define parallel pathways for small RNA biogenesis. Dicer-1 (Dcr-1) liberates miRNA from pre-miRNA, whereas Dicer-2 (Dcr-2) excises small interfering RNA (siRNA) from long double-stranded RNA (dsRNA) [21–23]. Like Drosha, *Drosophila* Dcr-2 requires a dsRBD partner protein, R2D2, for its function in siRNA biogenesis. Unlike Drosha, Dcr-2 suffices to process its substrate. However, without R2D2, Dcr-2 cannot load the siRNAs it produces into the RNA-induced silencing complex (RISC), the RNA interference (RNAi) effector complex [21,24,25]. Although born as small RNA duplexes, both siRNA and miRNA function in RISC as single-stranded RNA guides for members of the Argonaute family of proteins [26–28]. Among the five *Drosophila* Argonaute proteins, two—Ago1 and Ago2—are required for small RNA-directed cleavage of target RNAs [29]. In fact, the Piwi domain of Argonaute proteins is a structural homolog of the endoribonuclease RNase H, an enzyme that cleaves the RNA strand of DNA–RNA hybrids [30–33]. Both human and *Drosophila* Ago2 and *Drosophila* Ago1 provide the Mg^2+^-dependent catalytic subunit of RISC [29,31,34,35,36].

The *Drosophila* genome encodes three RNase III endonucleases, all of which act in miRNA or siRNA biogenesis. Whereas both Drosha and Dcr-2 associate with dsRBD proteins that facilitate their functions, no dsRBD protein partner has been assigned to Dcr-1. We asked if Dcr-1 might also partner with a dsRBD protein. Here, we identify the dsRBD protein Loquacious (Loqs), a paralog of R2D2, as the partner of Dcr-1. Mutation of *loqs* in flies and depletion of *loqs* in Schneider-2 (S2) cells by dsRNA-triggered RNAi disrupt normal pre-miRNA processing. In vivo, *loqs* is required for robust miRNA-directed silencing and complete target gene repression directed by a transgene expressing dsRNA. Moreover, loss of Loqs function in the ovary disrupts germ-line stem cell maintenance, rendering *loqs* mutant females sterile.

## Results

**Figure 1:**
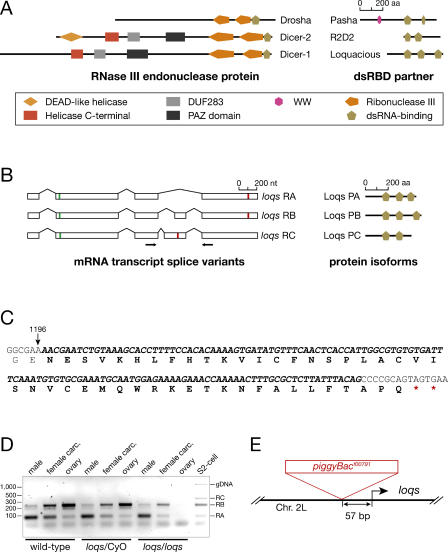
Loqs, a dsRBD Partner Protein for *Drosophila* Dcr-1 (A) Each of the three *D.* *melanogaster* RNase III endonucleases pairs with a different dsRBD protein, which assists in its function in RNA silencing. (B) Differential splicing creates three *loqs* mRNA variants, *loqs* RA, RB, and RC. *loqs* RA and RB are reported in FlyBase. The RC splice variant is reported here. Arrows mark the position of the PCR primers used in (D); green lines, start codons; red lines, stop codons. The resulting protein isoforms are diagrammed to the right. (C) Use of an alternative splice acceptor site extends the 5′ end of exon 4. The mRNA sequence surrounding the new exon–exon junction is shown, with the *loqs* RC-specific sequence in bold; the arrow marks the position of the last nucleotide of exon 3 relative to the putative transcription start site. When translated into protein, the exon 4 extension inserts 43 new amino acids (indicated below the mRNA sequence) and shifts the Loqs PC reading frame, truncating the protein. (D) RT-PCR analysis of *loqs* mRNA species in males, female carcasses remaining after ovary dissection, dissected ovaries, and S2 cells. Males express more *loqs* RA than *loqs* RB, female somatic tissue expresses both *loqs* RA and *loqs* RB, while ovaries express predominantly *loqs* RB. *loqs* RC was observed only in S2 cells, together with *loqs* RA and *loqs* RB. (E) The piggyBac transposon insertion f00791 lies 57 bp upstream of the reported transcription start site for *loqs*.

To identify a dsRBD protein partner for Dcr-1, we searched the conserved domain database [37] for all *Drosophila* proteins that contain dsRBDs. The protein encoded by the gene CG6866 has two dsRBDs, which are most closely related to dsRBD 1 and 2 of R2D2, suggesting that the two genes are paralogs (Figure 1A). CG6866 and R2D2 are 37% similar and 25% identical in the region of the two dsRBDs. A third dsRBD at the C-terminus of CG6866 was detected using the PFam collection of protein sequence motifs. This truncated domain deviates from the canonical dsRBD sequence. Because loss of CG6866 function de-silences both endogenous silencing and reporter expression in vivo (below), we named the gene *loquacious (loqs).* *loqs* is located on the left arm of Chromosome 2 at polytene band 34B9. *loqs* produces at least three different mRNA isoforms through alternative splicing (Figure 1B). The shortest transcript, *loqs* RNA splice variant A (RA), encodes a 419-amino-acid protein, Loqs protein isoform A (PA), with a predicted molecular mass of 45 kDa. The transcript *loqs* RNA splice variant B (RB) contains one additional exon and encodes a protein of 465 amino acids, Loqs protein isoform B (PB), with a predicted molecular mass of 50 kDa. These two mRNA species were identified as cDNAs in the *Drosophila* genome sequencing project and annotated in FlyBase [38] among the *Drosophila* proteins that contain dsRBDs. Using non-quantitative RT-PCR, we detected a third splice variant, *loqs* RNA splice variant C (RC), in which an alternative splice acceptor site for exon 4 is used (Figure 1B, C, and D). Use of the alternative splice site creates a 5′-extended fourth exon and changes the reading frame, resulting in a truncated protein, Loqs protein isoform C (PC), 383 amino acids long (Figure 1C). Loqs PC has a predicted molecular mass of 41 kDa and lacks the entire third dsRBD of Loqs PA and PB (Figure 1B). *loqs* RA is the predominant mRNA species in dissected testes, whereas *loqs* RB is the most abundant species in ovaries. Both isoforms are expressed in the carcasses of males and females after removal of the gonads (Figure 1D and data not shown). Using two independent antibodies raised against an N-terminal Loqs peptide, but not using pre-immune sera, we detected a candidate protein for Loqs PC in S2 cells (see below), suggesting that the three *loqs* transcripts give rise to distinct Loqs protein isoforms.

Thibault and co-workers reported a mutant allele of CG6866, *loqs* ^f00791^, recovered in a large-scale piggyBac transposon mutagenesis screen of *Drosophila* [39]. The f00791 piggyBac inserted 57 nucleotides upstream of the *loqs* transcription start site (Figure 1E); although annotated as lethal, homozygous mutant *loqs* ^f00791^ flies are viable but completely female sterile. Precise excision of the f00791 piggyBac transposon fully reverted the female sterility (data not shown). Analysis by quantitative RT-PCR using primers that amplify all three *loqs* mRNA splice variants (see Materials and Methods) showed that somatic female *loqs* ^f00791^ tissues express approximately 5-fold (4.76 ± 0.24; *n* = 3) less *loqs* mRNA than wild-type, while *loqs* ^f00791^ mutant ovaries express approximately 40-fold (42 ± 0.33; *n* = 3) less *loqs* mRNA than wild-type ovaries. Testes express approximately 3-fold (2.9 ± 0.5; *n* = 3) less *loqs* mRNA in the *loqs* ^f00791^ mutant than in wild type. These data suggest that the mutant phenotype should be strongest in ovaries, consistent with the mutation causing female sterility as its most obvious defect.

### In Vivo, Normal Pre-miRNA Processing Requires Loqs

**Figure 2:**
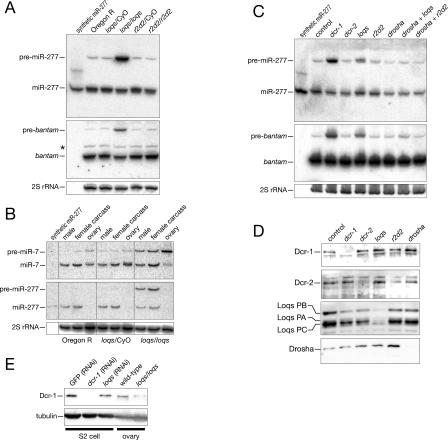
Loss of Loqs Function Increases the Steady-State Concentration of Pre-miRNA (A) Northern analysis of total RNA from wild-type, *loqs* ^f00791^ heterozygotes and homozygotes, and *r2d2* heterozygotes and homozygotes for whole males, probed for miR-277 and *bantam*. The membrane was first hybridized with the miR-277 probe, stripped and probed for 2S rRNA as a loading control, then stripped again and probed for *bantam* miRNA. Asterisk: the 2S probe was not completely removed before the hybridization with the *bantam* probe, resulting in an additional band above the mature *bantam* RNA. (B) Total RNA from whole males, female carcasses remaining after ovary dissection, and dissected ovaries was probed for miR-7. As a control for successful dissection, the blot was also probed for miR-277, which is not expressed in ovaries (KF and PDZ, unpublished results). 2S rRNA again served as a loading control. (C) Depletion of *dcr-1* or *loqs* in S2 cells by RNAi leads to pre-miRNA accumulation. Total RNA was isolated after dsRNA-triggered RNAi of the indicated genes. The control sample was treated with dsRNA corresponding to the polylinker sequence of pLitmus28i. (D) Depletion of Dcr-1, Dcr-2, Loqs, and Drosha was confirmed by Western blotting. (E) Western blotting analysis demonstrates that Dcr-1 levels are not significantly reduced by depletion of Loqs by RNAi in S2 cells, but are lower in *loqs* ^f00791^ mutant ovaries.

To assess the function of *loqs* in miRNA biogenesis, we isolated total RNA from *loqs* ^f00791^ males and determined the steady-state levels of mature and pre-miRNA for miR-277 and *bantam* (Figure 2A), which are both expressed in adult tissues. We detected a 100-fold increase in pre-miR-277 and a 12-fold increase in pre-*bantam* RNAs in homozygous mutant *loqs* ^f00791^ males, but not in heterozygous *loqs* ^f00791^ or heterozygous or homozygous *r2d2* mutant males. In contrast, the amount of mature miR-277 or *bantam* was only slightly reduced in the *loqs* ^f00791^ homozygotes.

Since *loqs* mRNA expression is lowest in the ovaries of *loqs* ^f00791^ mutant flies, we analyzed the levels of pre-miR-7 and mature miR-7, a miRNA that is expressed in whole males, manually dissected ovaries, and the female carcasses remaining after removing the ovaries (Figure 2B). While pre-miR-7 increased in all *loqs* ^f00791^ homozygous mutant tissues, relative to wild-type or *loqs* heterozygotes, the disruption of miR-7 production in ovaries was striking: not only did pre-miR-7 accumulate, but also mature miR-7 was dramatically reduced. These data suggest that Loqs protein function is required for the maturation of miRNA and demonstrate a direct correlation between *loqs* mutant allele strength and disruption of miRNA processing.

### Loqs Is Required for Pre-miRNA Processing in *Drosophila* S2 Cells

To confirm the function of *loqs* in pre-miRNA processing, we depleted cultured *Drosophila* S2 cells of *loqs* mRNA by RNAi (Figure 2C). Eight days after incubating S2 cells with dsRNA corresponding to the first 300 nucleotides of the *loqs* coding sequence, we determined the steady-state levels of pre-miRNA and mature miRNA for miR-277 and *bantam*. Relative to an unrelated dsRNA control, dsRNA corresponding to *dcr-1* caused a approximately 9-fold and approximately 23-fold increase in steady-state pre-miR-277 and *bantam* levels, respectively, and dsRNA corresponding to *loqs* caused a approximately 2-fold and approximately 6-fold increase in steady-state pre-miR-277 and *bantam* levels, respectively. In these experiments, RNAi of *dcr-1* more completely depleted Dcr-1 protein than RNAi of *loqs* reduced Loqs protein (Figure 2D). RNAi of *dcr-2,* *r2d2,* or *drosha* did not alter pre-miRNA levels for either miR-277 or *bantam,* nor did it alter Dcr-1 or Loqs levels. The Drosha/Pasha protein complex functions before pre-miRNA processing, converting primary miRNA (pri-miRNA) to pre-miRNA. Consistent with the idea that Loqs functions with Dcr-1 to convert pre-miRNA to mature miRNA, RNAi of *drosha* together with *loqs* alleviated the high pre-miRNA levels observed for RNAi of *loqs* alone, demonstrating that Loqs acts after Drosha.

**Figure 3:**
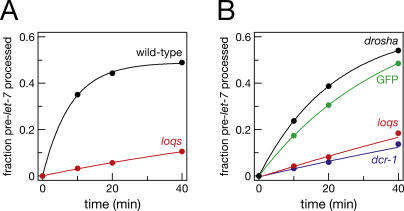
Loqs Is Required for Efficient pre-*let*-*7* Processing In Vitro (A) *loqs* ^f00791^ mutant ovary lysates processed pre-*let-7* into mature *let-7* miRNA ∼19-fold more slowly than wild-type. The data were fit to a first-order exponential equation, and initial velocities calculated from the fitted curve. (B) Analysis of pre-*let-7* processing in extracts from S2 cells. The cells were treated twice with dsRNA corresponding to the indicated genes.

Next, we examined processing of 20 nM exogenous pre-*let-7* into mature *let-7* in lysates from ovaries or S2 cells (Figure 3). Initial velocities were calculated for each reaction to permit comparison of processing rates (see Materials and Methods). Lysate from homozygous *loqs* ^f00791^ mutant ovaries processed pre-*let-7* RNA to mature *let-7* approximately 19-fold more slowly than wild-type ovary lysate (Figure 3A). Moreover, lysate prepared from S2 cells soaked with a green fluoresecent protein (GFP) control dsRNA (GFP[RNAi]) or *drosha* dsRNA (*drosha*[RNAi]) accurately and efficiently converted exogenous pre-*let-7* RNA into mature *let-7*. In contrast, both*dcr-1*(RNAi) and *loqs*(RNAi) S2 cell lysates converted pre-miRNA to mature miRNA approximately 5- and approximately 4-fold, respectively, more slowly than the control lysate (Figure 3B). Thus, Loqs is required for production in vivo of normal levels of miR-7, miR-277, and *bantam,* and the efficient conversion of pre-*let-7* to mature *let-7* in vitro. Together, these four miRNAs include both miRNAs found on the 5′ and on the 3′ side of the pre-miRNA stem, suggesting a general role for Loqs in pre-miRNA processing.

Reduction of R2D2 protein by RNAi destabilizes Dcr-2; conversely, RNAi of Dcr-2 renders R2D2 unstable [21]. In contrast, RNAi of *loqs* in S2 cells reduced Dcr-1 protein levels by no more than 15% (Figure 2D and E), suggesting that Loqs functions together with Dcr-1 in pre-miRNA processing, rather than that Loqs is simply needed to stabilize Dcr-1 protein. However, *loqs* ^f00791^ mutant ovaries, which lack detectable Loqs protein, contain 70% less Dcr-1 than wild-type (Figure 2E). A role for Loqs in both Dcr-1 function and in Dcr-1 stability suggests that the two proteins physically interact, like R2D2 and Dcr-2. Therefore, we tested if Dcr-1 and Loqs are components of a common complex.

### A Dcr-1 Protein Complex Contains Loqs

**Figure 4:**
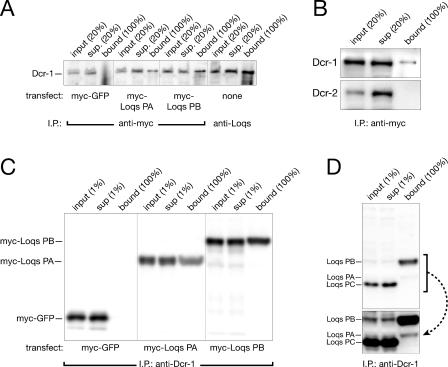
Loqs and Dcr-1 Are Present in a Common Protein Complex in S2-Cells (A) Dcr-1 associates with myc-tagged Loqs PA or PB, and with endogenous Loqs protein. Immunoprecipitation with anti-myc or anti-Loqs antibody was performed using lysates from S2 cells transfected with the indicated expression plasmid. Dcr-1 was detected by Western blotting. (B) myc-tagged Loqs PB stably associates with Dcr-1 but not Dcr-2. S2 cells were transfected with plasmid expressing myc-tagged Loqs PB, then lysed and immunoprecipitated with anti-myc antibody. The immunoprecipitates were analyzed by Western blotting using anti-Dcr-1 or anti-Dcr-2 antibodies. (C) S2 cells were transfected with plasmid expressing myc-tagged GFP, Loqs PA, or Loqs PB, then extracted and immunoprecipitated with anti-Dcr-1 antibody. The immunoprecipitates were analyzed by Western blotting using anti-myc antibody. (D) Anti-Dcr-1 antibody was used to immunoprecipitate Dcr-1 and associated proteins from S2 cell lysates, and the immunoprecipitates were analyzed by Western blotting using anti-Loqs antibody to detect endogenous Loqs protein. The major Loqs protein isoform recovered was Loqs PB. In a longer exposure (bottom panel), a band corresponding in size to Loqs PA is visible. The most abundant Loqs isoform the input sample, Loqs PC, which lacks the third dsRBD, did not immunoprecipitate with Dcr-1, suggesting that the third dsRBD is required for the association of Loqs with Dcr-1.

We expressed in S2 cells myc-tagged versions for two protein isoforms of Loqs, Loqs PA and Loqs PB, and immunoprecipitated the tagged proteins with anti-myc monoclonal antibodies. We analyzed the immunoprecipitated protein by Western blotting using a polyclonal anti-Dcr-1 antibody. Figure 4A shows that Dcr-1 protein co-immunoprecipitated with myc-tagged Loqs. When myc-tagged GFP was expressed in place of myc-tagged Loqs, no Dcr-1 protein was recovered in the anti-myc immunoprecipitate. Similarly, an affinity purified, polyclonal antibody directed against the N-terminus of endogenous Loqs protein also co-immunoprecipitated Dcr-1 protein (Figure 4A). This interaction was resistant to treatment with RNase A (data not shown). We could not detect co-immunoprecipitation of Dcr-2 with myc-tagged Loqs PB under conditions where Dcr-1 was readily detected (Figure 4B), but we cannot exclude that Dcr-2 is a substoichiometric component of a complex that contains both Dcr-1 and Loqs (see below).

When immunoprecipitated with anti-Dcr-1 antibody, both myc-tagged Loqs protein isoforms—PA and PB—associated with Dcr-1 (Figure 4C). Moreover, the antibody against endogenous Loqs protein detected two bands corresponding in size to Loqs PA and Loqs PB in the proteins immunoprecipitated with the anti-Dcr-1 antibody (Figure 4D). Loqs PB comprises only approximately 22% of the total Loqs protein in S2 cells, but corresponded to approximately 95% of the Loqs associated with Dcr-1. Loqs PA, which is expressed at comparable levels in S2 cells, accounts for most of the remaining Loqs associated with Dcr-1. In contrast, the putative Loqs PC protein comprises the majority of S2 cell Loqs, but was not recovered in the Dcr-1 immunoprecipitate. Intriguingly, Loqs PA and PB contain a third dsRBD that Loqs PC lacks; perhaps this third dsRBD is required for the association of Loqs with Dcr-1.

**Figure 5:**
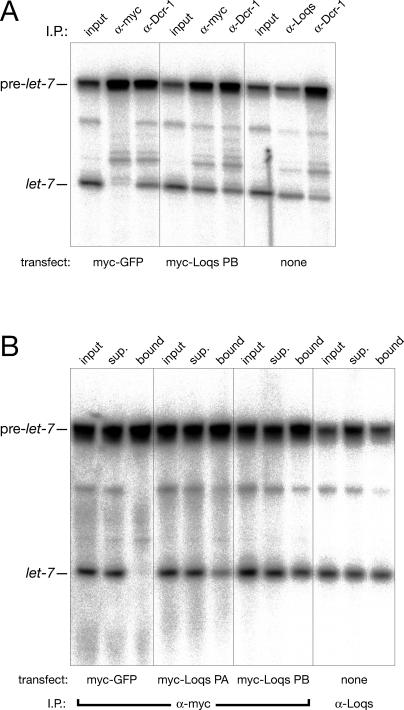
Loqs Is Associated with Pre-miRNA Processing Activity in S2 Cells (A) Pre-miRNA processing activity co-immunoprecipitates with myc-tagged Loqs PB and with endogenous Dcr-1 or endogenous Loqs, but not with myc-tagged GFP. (B) Pre-miRNA processing activity co-purifies by immunoprecipitation with both Loqs protein isoforms that interact with Dcr-1, Loqs PA, and Loqs PB. The extracts used in (A) and (B) were independently prepared.

The immunoprecipitated Dcr-1–Loqs complexes accurately converted pre-miRNA to mature miRNA (Figure 5). Pre-miRNA processing by the immunoprecipitates was efficient and accurate when we used the anti-Dcr-1 antibody (Figure 5A), and when we used anti-myc antibody and expressed myc-tagged Loqs, but not when we used the anti-myc antibody and expressed myc-tagged GFP (Figure 5A and 5B). Thus, Dcr-1 and Loqs co-associate in a complex capable of converting pre-miRNA into mature miRNA. Our data also demonstrate that an N-terminal tandem myc tag does not perturb Loqs function in pre-miRNA cleavage.

**Figure 6:**
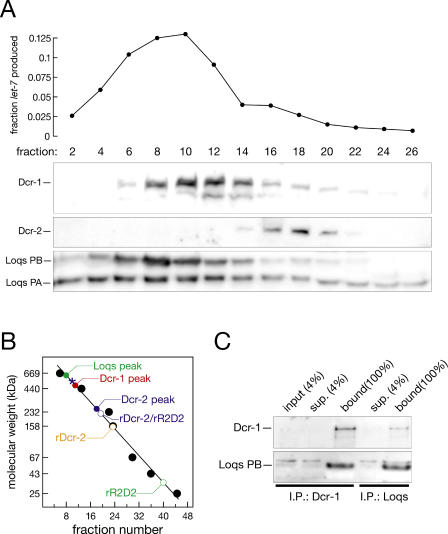
Analysis of Complexes Containing Pre-miRNA Processing Activity, Dcr-1, and Loqs (A) S2 cell lysate was fractionated by gel filtration chromatography and analyzed for pre-*let-7* processing activity, and Dcr-1, Dcr-2, and Loqs proteins. (B) The sizes of the distinct complexes containing Loqs (∼630 kDa), Dcr-1 (∼480 kDa), and Dcr-2 (∼230 kDa) and the broad complex containing pre-miRNA processing activity (∼525 kDa) were estimated using molecular weight standards (thyroglobulin, 669 kDa; ferritin, 440 kDa; catalase, 232 kDa; aldolase, 158 kDa; bovine serum albumin, 67 kDa; ovalbumin, 43 kDa; chymotrypsinogen A, 25 kDa) and recombinant Dcr-2 and R2D2 proteins (rDcr-2 and rR2D2). The blue asterisk denotes the peak of pre-*let-7* processing activity detected in (A). (C) Fractions containing the Dcr-1 peak were pooled and immunoprecipitated with either anti-Dcr-1 or anti-Loqs antibodies. Western blotting with anti-Dcr-1 and anti-Loqs antibodies demonstrated that Dcr-1 and Loqs remained associated through gel filtration chromatography.

Next, we estimated the size of the pre-miRNA processing complex by gel filtration chromatography. Pre-miRNA processing activity chromatographed as a broad approximately 525-kDa peak that overlapped the peaks of both Dcr-1 and Loqs proteins (Figure 6A and 6B). Dcr-1 protein chromatographed as an approximately 480-kDa complex that overlapped the peak of Loqs PB, which chromatographed as an approximately 630-kDa complex. The Loqs PB isoform accounts for most of the Dcr-1-associated Loqs in S2 cells (see Figure 4D). The apparent size of the Dcr-1 complex suggests that it is either associated with proteins in addition to Loqs or that the complex has an elongated shape that increases its apparent molecular weight. Pre-miRNA processing activity, Loqs, and Dcr-1 were all well resolved from the approximately 230-kDa peak of Dcr-2 (theoretical mass = 197.7 kDa), which corresponds to the Dcr-2/R2D2 heterodimer (theoretical mass = 232.7 kDa). Although the peaks of Loqs and Dcr-1 do not co-migrate, Dcr-1 was stably associated with Loqs after gel filtration: Dcr-1 and Loqs reciprocally co-immunoprecipitated from the pooled peak Dcr-1 fractions (Figure 6C). Loqs was not detected in the Dcr-2 peak by this method (data not shown). Loqs PC, which did not associate with Dcr-1 in immunoprecipitation, chromatographed as a 58-kDa protein, suggesting that it is a free monomeric protein (data not shown).

### A Loqs Mutation Reduces Silencing of a miRNA-Controlled Reporter Transgene In Vivo

**Figure 7:**
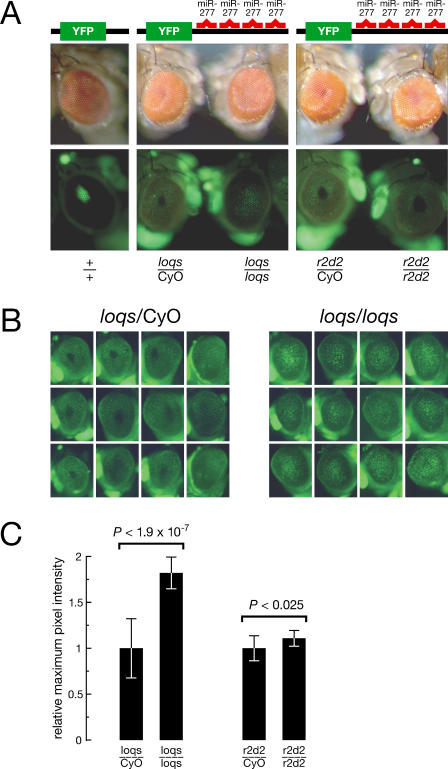
Silencing of a miRNA-Responsive YFP Reporter Requires *loqs* but Not *r2d2* (A) A YFP transgene expressed from the Pax6-promoter showed strong fluorescence in the eye and weaker fluorescence in the antennae. Due to the underlying normal red eye pigment, the YFP fluorescence was observed in only those ommatidia that are aligned with the optical axis of the stereomicroscope. In heterozygous *loqs* ^f00791^/CyO flies bearing a miR-277-responsive, Pax6-promotor-driven, YFP transgene, YFP fluorescence was visible in the antennae but was repressed in the eye. In contrast, in homozygous mutant *loqs* ^f00791^ flies, YFP fluorescence was readily detected in the eye. A strong mutation in *r2d2* did not comparably alter repression of the miR-277-regulated YFP reporter. The exposure time for the unregulated YFP reporter strain was one-fourth that used for the miR-277-responsive YFP strain. The exposure times were identical for the heterozygous and homozygous *loqs* and *r2d2* flies. (B) Additional images of eyes from *loqs* ^f00791^ heterozygous and homozygous flies bearing the miR-277-responsive YFP reporter transgene diagrammed in (A). (C) Quantification of fluorescence of the miR-277-responsive YFP transgene in eyes heterozygous or homozygous for *loqs* or *r2d2*. The maximum pixel intensity was measured for each eye (excluding antennae and other tissues where miR-277 does not appear to function). The graph displays the average (*n* = 13) maximum pixel intensity ± standard deviation for each homozygous genotype, normalized to the average value for the corresponding heterozygotes. Statistical significance was estimated using a two-sample Student's *t*-test assuming unequal variance. The images in (A) were acquired using a sensitive, GFP long-pass filter set that transmits yellow and red autofluorescence. Images in (B) and for quantitative analysis were acquired using a YFP-specific band-pass filter set that reduced the autofluorescence recorded.

**Figure 8:**
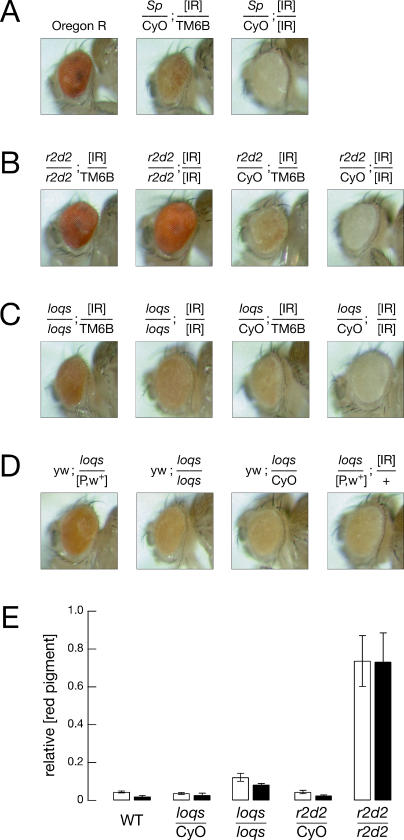
Silencing of *white* by an IR Partially Depends on *loqs* (A) The red eye color of wild-type flies (left) changes to orange (center) and white (right) in response to one or two copies, respectively, of a *white* IR transgene, which silences the endogenous *white* gene. (B) Homozygous mutant *r2d2* flies fail to silence *white,* even in the presence of two copies of the *white*-IR transgene; heterozygous *r2d2*/CyO flies repress *white* expression. (C) In flies homozygous for *loqs* ^f00791^, silencing of *white* by the *white*-IR is less efficient; two copies of the *white*-IR do not produce completely white eyes, whereas they do in heterozygous *loqs* ^f00791^/CyO. (D) The eye color change in *loqs* ^f00791^ flies is not caused by the increased *white^+^* gene dose resulting from the mini*-white* marker in the *piggyBac* transposon that causes the *loqs* ^f00791^ mutation. Flies *trans*-heterozygous for *loqs* ^f00791^ and a mini-*white*-marked P-element have more red eye pigment than *loqs* ^f00791^ homozygous flies, but show more efficient silencing by the *white*-IR than *loqs* ^f00791^ homozygous animals. (E) The eye pigment of the indicated genotypes was extracted and quantified by green light (480 nm) absorbance, relative to wild-type flies bearing no *white*-IR transgenes. The graph shows the mean and standard deviation of five independent measurements per genotype.

The *loqs* ^f00791^ mutation caused pre-miRNAs to accumulate in the soma and the germ line and strongly reduced mature miR-7 levels in the female germ line, suggesting that Loqs function is required for miRNA-directed silencing in vivo. We introduced a miRNA-regulated yellow fluorescent protein (YFP) reporter into *loqs* ^f00791^ homozygous mutant flies. This transgenic reporter expresses in the eye a YFP mRNA bearing four miR-277 binding sites in its 3′ UTR. The four miRNA-binding sites pair with all but the central three nucleotides of miR-277 and are, therefore, predicted to repress reporter mRNA translation rather than trigger mRNA cleavage (Figure 7A). YFP fluorescence was readily detected in the eye and antennae in control flies in which the 3′ UTR of the YFP transgene lacked the four miR-277 binding sites (Figure 7A). When the reporter contained the miR-277 binding sites, YFP expression was repressed in the eye but readily visible in the antennae, indicating that miR-277 is expressed in the eye (*loqs*/CyO, Figure 7A and B). This expression was verified independently by Northern blots of RNA isolated from eyes dissected away from other tissues of the head (data not shown). Silencing of the miR-277-responsive YFP reporter in the eye was reduced in *loqs* ^f00791^ homozygous mutant flies (*loqs*/*loqs*, Figure 7A, B and C). As a control, we examined the effect of a strong *r2d2* mutation on YFP reporter expression (Figure 7A and C). We measured the maximum fluorescence intensity in each eye for all four genotypes. Figure 7C shows that there was a significant (*P* < 1.9 × 10^−7^) increase in YFP fluorescence in eyes homozygous for the weak hypomorphic allele *loqs* ^f00791^. This allele reduced miR-277 levels in the soma approximately 2-fold (see Figure 2B); fluorescence in the eye of homozygous mutant *loqs* flies was 1.8 ± 0.17 (average maximum intensity ± standard deviation; *n* = 13) times greater than in the eyes of their age-matched heterozygous siblings. In contrast, flies homozygous for a strong hypomorphic *r2d2* mutation show only a modest change in fluorescence (1.1 ± 0.09; *n* = 13; *P* < 0.025). The Dcr-2 partner protein R2D2 is required for RNAi triggered by exogenous dsRNA [21] or transgenes expressing long dsRNA hairpins (see below and Figure 8). We conclude that the reduced levels of Loqs protein in the *loqs* ^f00791^ mutant lead to a statistically significant reduction in miRNA-directed silencing and that the Loqs paralog R2D2 plays little, if any, role in miRNA function.

### Loqs Participates in Silencing Triggered by Long dsRNAIn Vivo

dsRNA transcribed as an inverted repeat (IR) triggers silencing of corresponding mRNAs in flies [23,40]. For IR-silencing of the *white* gene, whose gene product is required to produce the red pigment that colors fly eyes, the extent of silencing is proportionate to the number of copies of the IR-*white* transgene [23,40] (Figure 8A), but is relatively insensitive to the number of copies of *white* present (TD and PDZ, unpublished). RNAi in *Drosophila* requires both Dcr-2, which transforms long dsRNA into siRNA, and R2D2, which collaborates with Dcr-2 to load siRNA into RISC. Thus, IR-silencing of *white* mRNA is lost in both *dcr-2* [23] and *r2d2* mutant flies (Figure 8B). We quantified the extent of *white* silencing by extracting the eye pigment in acidic ethanol and measuring its absorbance at 480 nm (Figures 8E and S1). Loss of R2D2 function in flies expressing one (or two) copies of the *white* IR transgene and two copies of the endogenous *white* locus restored red pigment levels to 74 ± 13 (or 73 ± 15 for two copies of IR-*white*) percent of wild-type flies lacking the *white*-IR. *loqs* ^f00791^ mutant flies were also defective in IR-triggered *white* silencing, but to a much smaller extent (Figure 8C and 8E). The *loqs* ^f00791^ mutation restored pigment levels in flies carrying one copy of the *white* IR-expressing transgene to 12 ± 2% of wild-type and to 8 ± 0.6% for flies carrying two copies of the *white*-IR (*n* = 5; Figures 8C and E). *loqs* ^f00791^ heterozygotes were statistically indistinguishable from wild-type flies bearing one copy of IR-*white*, whose eye pigment concentration was 4 ± 0.5 (or 2 ± 0.6 for two copies of IR-*white*) percent of wild-type in the absence of the IR-*white* transgene.

Insertion of a mini-*white*-expressing piggyBac transposon causes the *loqs* ^f00791^ allele. Thus, *loqs* ^f00791^ heterozygotes have two copies of the endogenous *white* locus and one copy of mini-*white*; *loqs* ^f00791^ homozygotes have two copies of endogenous *white* and two copies of mini-*white*. The presence of this additional copy of mini-*white* does not account for the darker red color of *white*-silenced *loqs* ^f00791^ flies, because *loqs* ^f00791^ heterozygotes bearing two copies of *white,* one copy of mini-*white* (in the piggyBac transposon inserted at *loqs*), and one copy of a P-element expressing mini-*white* are effectively silenced by IR-*white* (Figure 8D). In the absence of the IR-*white* transgene, the total amount of *white* expression in these flies is higher than in *loqs* ^f00791^ homozygotes (Figure 8D). Thus, reduction of Loqs function accounts for the partial desilencing of *white* in this system. The modest loss of silencing in the *loqs* ^f00791^ mutant flies may reflect the incomplete loss of Loqs protein in this allele. However, Carthew and co-workers previously reported that a *dcr-1* null mutation leads to a similar, partial loss of *white* IR-silencing [23]. The small eye phenotype of *dcr-1* null mutants unfortunately renders a quantitative comparison to *loqs* ^f00791^ impossible. We propose that—as for pre-miRNA processing—Dcr-1 and Loqs act together to enhance silencing by siRNAs.

### Silencing of the Endogenous Stellate Locus Requires Loqs

**Figure 9:**
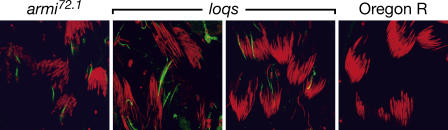
Silencing of *Stellate* by the dsRNA-Generator *Su(Ste)* Requires *loqs* Testes were stained for DNA (red) and Stellate protein (green). Defects in RNA silencing often lead to accumulation of Stellate protein crystals in testes. For example, the testes from the strong allele *armi^72.1^,* but not wild-type Oregon R testes, show Stellate protein staining. Testes from *loqs* ^f00791^ males show strong accumulation of Stellate protein, consistent with their significantly impaired fertility.

*loqs* ^f00791^ males are incompletely fertile. When Oregon R females were mated to *loqs* ^f00791^ homozygous mutant males, only 17% of embryos hatched (*n* = 479); for *loqs* ^f00791^ heterozygous males, 47% of embryos hatched (*n* = 466). Ninety percent of embryos hatched (*n* = 753) for wild-type Oregon R males. Genes required for RNA silencing often reduce male fertility, because the X-linked gene*Ste* is epigenetically silenced in testes by dsRNA derived from the bi-directionally transcribed *Suppressor of Stellate (Su(Ste))* locus [41]. *Ste* silencing is genetically similar, but not identical, to RNAi, in that like RNAi it requires the function of the gene *armitage* *(armi)* [24], but unlike RNAi does not require *r2d2* (VVV and PDZ, unpublished data). In the absence of *Ste* silencing, Stellate protein accumulates as protein crystals in the testes. *loqs* ^f00791^ mutants contain Stellate crystals in their testes (Figure 9), much like *armi^72.1^* mutants, identifying a second role for *loqs* in silencing by endogenous RNA triggers, distinct from its function in miRNA biogenesis.

### A Germ-Line Stem Cell Defect in *loqs* ^f00791^ Mutant Females

**Figure 10:**
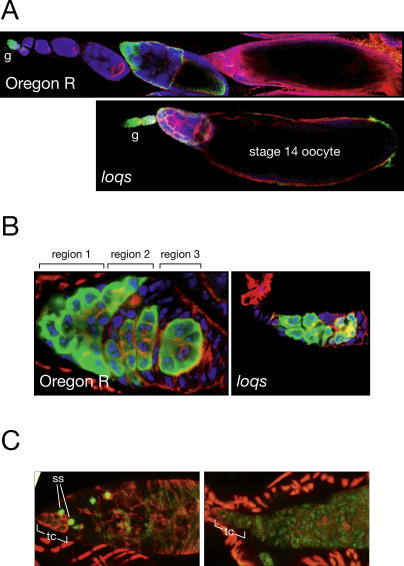
loqs^f00791^ Fail to Maintain Germ-Line Stem Cells (A) Wild-type ovarioles contain a germarium and a developmentally ordered array of six to eight egg chambers, whereas *loqs* ^f00791^ mutant ovarioles contain a smaller than normal germarium, two or three pre-vitellogenic egg chambers, and a late-stage egg chamber. Wild-type and *loqs* ovarioles are shown at the same magnification. (B) In wild-type ovarioles, the germarium contains several newly formed germ-line cysts surrounded by somatic follicle cells. In contrast, *loqs* ^f00791^ mutant germaria contain few germ-line cells, which are not organized into distinct cysts. The follicle cell layer is also significantly reduced in*loqs* ^f00791^ germaria. (C) Wild-type and *loqs* mutant germaria labeled for α-Spectrin (green) and filamentous Actin (red). In wild type, anti-α-Spectrin labels the spectrosome (ss), a structure characteristic of germ-line stem cells, which are normally found at the anterior of the germarium, apposed to the somatic terminal cells (tc). The cystoblasts, the daughters of the stem cells, also contain a spectrosome, but are located posterior to the stem cells. In *loqs* mutant ovaries, spectrosome-containing cells were not detected, indicating that normal germ-line stem cells are not present. These observations indicate that stem cells are not maintained. In (A) and (B), ovaries were labeled for filamentous actin (red) using rhodamine phalloidin, DNA (blue) using TOTO3 (Molecular Probes), and the germ-line marker Vasa (green) using rabbit anti-Vasa antibody detected with fluorescein-conjugated anti-rabbit secondary antibody. In (B) and (C), wild-type and *loqs* germaria are shown at the same magnification.

The *loqs* gene has a critical function in oogenesis, as *loqs* ^f00791^ females have small ovaries (Figure 10A) and are completely sterile. *Drosophila* ovaries comprise ovarioles that contain developmentally ordered egg chambers, which are produced continuously in the adult by germ-line stem cell division. As a result, mutations that block stem cell division or maintenance lead to ovarioles containing few egg chambers. *loqs* ^f00791^ mutant females lay no eggs. Whereas wild-type females contain 7 ± 0.8 (*n* = 15) previtellogenic egg chambers per ovariole, *loqs* ^f00791^ contain only 3 ± 0.8 (*n* = 20). Excision of the *piggybac* transposon in *loqs* ^f00791^ restores fertility, demonstrating that these defects reflect loss of Loqs function. The mature oocytes in *loqs* ^f00791^ ovarioles have normal dorsal appendages, indicating that dorsoventral patterning is normal. In contrast, mutations in *armi, spnE,* and *aub* disrupt both dorsoventral and anteroposterior patterning [42–44]. These mutations all disrupt RNAi and*Ste* silencing, but display no global defects in miRNA biogenesis or function, unlike *loqs* [24,41,45,46].

Oogenesis is initiated in the germarium, which contains the germ-line stem cells as well as the early germ-line cysts that will form egg chambers. In *loqs* ^f00791^ mutant ovarioles, the germaria generally contain a limited number of cells that stain for Vasa, indicating that they are of germ-line origin (Figure 10B). No mitotic figures were observed, nor were separate cysts. Germ-line stem cells and their daughter cells, the cystoblasts, are characterized by the presence of a spherical structure, the spectrosome, that stains intensely with anti-Spectrin antibodies [47–49]. We stained wild-type and *loqs* ^f00791^ germaria with anti-α-Spectrin antibodies (Figures 10C and S2). We could not detect spectrosomes in the *loqs* mutant germaria, suggesting that in these germaria, dissected from flies 3–4 d old, no stem cells remained. Stem cells must have originally been present, because *loqs* mutant ovaries produce some late-stage oocytes. Thus, most of the original stem cells may have died or differentiated into cystoblasts without renewing the stem cell pool. At present, we cannot distinguish between these alternatives. We conclude that *loqs* ^f00791^ mutants, which are defective in three distinct types of RNA silencing, fail to maintain germ-line stem cells.

## Discussion

### RNase III Endonucleases Act with dsRNA-Binding Partner Proteins in RNA Silencing

Collectively, Dcr-1 and Loqs, Drosha and Pasha, and Dcr-2 and R2D2 comprise six of the 12 dsRBD proteins predicted to be encoded by the *Drosophila* genome [50]. Thus, at least half of all dsRBD proteins in flies participate in RNA silencing. In *Caenorhabditis elegans*, the R2D2-like dsRBD protein RDE-4 is required for RNA interference and interacts with DCR-1, the sole worm Dicer gene [51]. RDE-4 is equally similar to Loqs (E-value = 0.03) and R2D2 (E-value = 0.026; search restricted to *C. elegans* proteins). The Drosha/Pasha complex is also present in *C. elegans* [10] as well as cultured human cells [11–13]. Similarly, the *Arabidopsis thaliana* dsRBD protein HYL1 is required for the production of mature miRNAs, and *hyl1* mutant plants have a phenotype similar to that of *dicer-like 1 (dcl1)* [52,53]. Hiraguri and co-workers [54] recently demonstrated that HYL1 is a dsRNA-binding protein that binds DCL1 and that the HYL1 paralog DRB4 binds the Dicer protein DCL4. Pairing of RNase III endonucleases with dsRBD proteins is thus a recurring theme in RNA silencing.

### A dsRBD Partner for Human Dicer?

The human genome encodes one Dicer protein, which is more closely related to *Drosophila* Dcr-1 than Dcr-2. Sequence analysis of human proteins for similarity to either *C. elegans* RDE-4 or *Drosophila* R2D2 does not identify a reasonable candidate for a dsRBD partner protein for human Dicer. In contrast, the human TRBP is highly similar to *Drosophila* Loqs (E-value = 5 × 10^−36^). For comparison, the human proteins most similar to R2D2 or RDE-4 give E-values of 8 × 10^−8^ and 0.42, respectively, when the search is restricted to human proteins. Human TRBP was first identified [55] because it binds HIV *trans*-activator RNA (TAR), a stem-loop structure required for active HIV transcription [56–58]. Remarkably, the secondary structure of TAR resembles a miRNA precursor, and the recent discovery of Epstein-Barr virus-encoded miRNAs [59] has fueled speculation that TAR may be a viral pre-miRNA [60].

Deletion of PRBP, the mouse homolog of TRBP, yields viable mice that often die at the age of weaning. Surviving homozygous mutant males show defects in spermatogenesis attributed to abnormal sperm maturation rather than proliferation [61]. In contrast, Dicer knockout mice show very early embryonic lethality [62]. If mouse Dicer and PRBP collaborate to produce mature miRNA, the essential function of Dicer during mouse development must either be independent of miRNA function, or a redundant factor must replace PRBP during embryonic development but not spermatogenesis.

### Loqs and Dcr-1 Protein Complexes

Together with *dcr-1,* the gene *loqs* is required in flies for normal pre-miRNA processing. Loqs and Dcr-1 reciprocally co-immunoprecipate. Pre-miRNA processing activity also co-immunoprecipitates with Dcr-1 and Loqs. However, in gel filtration chromatography, the two proteins overlap but do not precisely co-purify. Loqs and Dcr-1 may form a protein complex analogous to the Dcr-2/R2D2 and Drosha/Pasha complexes [10,21], but this complex may be transient, with Loqs also associating with other components of the RNA silencing machinery, perhaps even escorting the mature miRNA to Ago1, an approximately 110-kDa Argonaute protein associated with mature miRNAs in flies. In fact, the predominant Loqs-containing complex in S2 cell lysate is about 150 kDa larger than the peak of Dcr-1, so it could contain Dcr-1, Loqs, and Ago1. The data of Siomi and co-workers demonstrating that Ago1 associates with both Dcr-1 [28] and Loqs (Saito K, et al. DOI: 10.1371/journal.pbio.0030235) support such a view.

### Cross-Talk between the Dcr-1 and Dcr-2 Pathways in *Drosophila*

In humans and *C. elegans,* a single Dicer gene is responsible for generating both siRNAs and miRNAs. *Drosophila* has apparently duplicated both its ancestral Dicer RNase III endonuclease and its dsRBD partner protein, dedicating Dcr-1/Loqs to miRNA processing and Dcr-2/R2D2 to RNAi. Nonetheless, these two pathways are not completely separate, because cells lacking*dcr-1* are not fully competent for IR-triggered silencing [23]. Dcr-1 is not required for siRNA production, yet embryo extracts lacking Dcr-1 fail to assemble RISC [23]. Dcr-1 has been proposed to be a component of “holo-RISC,” an 80S complex containing many, but not all, components of the RNAi pathway in flies [63]. The *loqs* ^f00791^ mutation also reduced the efficiency of IR-triggered silencing in vivo. Therefore, we propose that Dcr-1 must partner with Loqs not only during the processing of pre-miRNA to mature miRNA, but also to ensure Dcr-1 function in the Dcr-2-dependent RNAi pathway.

Carthew and colleagues found no function for Dcr-2 in miRNA biogenesis [23]. Consistent with their results, we found little if any requirement for R2D2 in miRNA-directed silencing (see Figure 7C). Moreover, null or strong hypomorphic alleles of either *dcr-2* or *r2d2* show no overt phenotype, whereas the *dcr-1^Q1147X^* null mutation is embryonic lethal [23].

### Stellate Silencing Requires Loqs

Endogenous silencing of the *Stellate* locus in testes is genetically distinct from miRNA-directed silencing, because it requires *armitage,* a gene that plays no general role in miRNA biogenesis or function [24]. *Stellate* silencing resembles RNAi in that *Stellate* expression is repressed by a dsRNA trigger transcribed from the *Su(Ste)* gene. *Su(Ste)* dsRNA produces siRNAs, called repeat-associated siRNAs, that are longer than the siRNAs produced in the RNAi pathway in *Drosophila* [41]. Even the weak allele described in this study, *loqs* ^f00791^ *,* which reduces *loqs* mRNA levels only approximately 3-fold in testes, dramatically de-silences *Stellate*. Given the intimate association of Dcr-1 with Loqs, our data raise the possibility that Loqs acts to silence *Stellate* in collaboration with Dcr-1, which may generate the *Su(Ste)* repeat-associated siRNAs.

### Germ-Line Stem Cells and miRNAs

The *loqs* ^f00791^ mutation is the first viable allele in *Drosophila* with a generalized defect in miRNA production. The allele may therefore be useful for future phenotypic analysis of miRNA-dependent pathways during the life cycle of *Drosophila*. The most obvious phenotype of *loqs* ^f00791^ is female sterility. *loqs* ^f00791^ homozygotes produce few egg chambers, indicating a defect in germ-line stem cell maintenance or division. The *loqs* ^f00791^ phenotype is similar to mutants in *piwi* [64], which encodes a member of the Argonaute protein family of core RISC components. In *piwi* mutant ovaries, germ-line stem cells fail to divide and instead differentiate directly into cystoblasts, depleting the germarium of germ-line stem cells. *loqs* mutants display a similar phenotype: we did not detect germ-line stem cells (i.e., spectrosome-containing cells) in *loqs* ^f00791^ homozygous germaria, suggesting that Loqs is required to maintain stem cells. Piwi is required in terminal filament cells, somatic cells surrounding the tip of the germarium, to send a signal that prevents germ-line stem cells from differentiating [64,65]. Piwi is also required in germ-line stem cells themselves to stimulate their proliferation [65]. Perhaps Piwi is at the core of an effector complex loaded with small RNA produced by Dcr-1 and Loqs. Intriguingly, *dcr-1* knockout mice die at embryonic day 7.5, apparently devoid of stem cells [62].

## Materials and Methods

### PiggyBac excision

To establish that insertion of the f00791 piggyBac transposon in the *loqs* gene caused the female sterility of *loqs* ^f00791^ mutants, we excised the transposon by introducing into *loqs* ^f00791^ heterozygotes a transgene expressing the piggyBac transposase from a Hermes element inserted on Chromosome 3 [66]. F1 male progeny of these flies were mated to *yw*; *Sp*/CyO virgins, and the resulting F2 progeny screened for loss of *white* expression (i.e., white eyes). Of 100 F2 progeny examined, one *white* ^-^ male was recovered. A line established from this fly was homozygous female fertile.

### Real-time RT-PCR analysis

Two μg of total RNA was reverse transcribed using 5′-GCG AAT TCT TTT TTT TTT TTT TTT TTT TTT-3′ oligonucleotide as primer and Superscript II reverse transcriptase (Invitrogen, Carlsbad, California, United States). After extension, cDNA samples were diluted 3-fold with water. One μl of diluted cDNA was used for quantitative PCR using the Quantitect SyBr-green kit (Qiagen, Valencia, California, United States) in a DNA Engine Opticon 2 (MJ Research [Bio-Rad, Hercules, California, United States]). Oligonucleotide primers were 5′-ATG GAC CAG GAG AAT TTC CAC GGC-3′ and 5′-GGC CTC GTC GCT GGG CAA TAT TAC-3′ for *loqs* and 5′-AAG TTG CTG CTC TGG TTG TCG-3′ and 5′-GCC ACA CGC AGC TCA TTG TAG-3′ for *actin5C*. Amplification efficiencies were identical for both oligonucleotide pairs.

### RNA isolation and detection by Northern blot

RNA was isolated from whole flies, dissected organs or S2 cells using Trizol (Invitrogen) according to the manufacturer's instructions. The RNA was quantified by absorbance at 260 nm, and 2–10 μg of total RNA was resolved by electrophoresis through a 20% denaturing acrylamide/urea gel (National Diagnostics, Atlanta, Georgia, United States). As a positive control for miR-277 hybridization, 10 fmol of phosphorylated miR-277 synthetic oligonucleotide (Dharmacon, Lafayette, Colorado, United States) was included on the gel. After electrophoresis, the gel was transferred to Hybond N+ (Amersham-Pharmacia, Little Chalfont, United Kingdom) in 0.5x TBE in a semi-dry transfer system (Transblot SD, Bio-Rad) at 20 V for 60 min. The RNA was UV cross-linked to the membrane (Stratalinker, Stratagene, La Jolla, California, United States) and pre-hybridized in 10 ml Church buffer [67] for 60 min at 37 °C.

RNA (Dharmacon) or DNA (IDT, Coralville, Iowa, United States) probes (25 pmol per reaction) were 5′-radiolabeled with polynucleotide kinase (New England Biolabs, Beverly, Massachusetts, United States) and γ-^32^P-ATP (New England Nuclear, Boston, Massachusetts, United States) (330 μCi per reaction; specific activity 7,000 Ci/mmol). After labeling, unincorporated radioactivity was separated from the labeled probe using a Sephadex G-25 spin column (Roche, Basel, Switzerland). The labeled probe oligonucleotide was added to 10 ml of Church buffer and used for hybridization. For RNA probes, hybridization was carried out at 65 °C; DNA probes were hybridized at 37 °C. For both, hybridization was overnight followed by five 30-min washes with 2× SSC/0.1% (w/v) SDS. Membranes were exposed to phosphorimaging screens (Fuji, Tokyo, Japan). To strip probes, the membranes were boiled twice in 0.1% SDS for 1 min in a microwave oven. The following probes were used for detection: 5′-UCG UAC CAG AUA GUG CAU UUU CA-3′ for miR-277; 5′-CAG CTT TCA AAA TGA TCT CAC T-3′ for bantam; 5′-ACA ACA AAA UCA CUA GUC UUC CA-3′ for miR-7; 5′-TAC AAC CCT CAA CCA TAT GTA GTC CAA GCA-3′ for 2S rRNA.

### Molecular cloning and generation of transgenic flies

Plasmids for the expression of myc-Loqs PA (pKF111) and myc-Loqs PB (pKF109) were created by PCR amplifying *loqs* mRNA with oligonucleotides 5′-AGC GGA TCC ATG GAA CAA AAA CTT ATT TCT GAA GAA GAC TTG GAA CAA AAA CTT ATT TCT GAA GAA GAC TTG GCC ATG GAC CAG GAG AAT TTC CAC GGC-3′ (appending two myc-tags to the N-terminus of Loqs) and 5′-TTA TGC GGC CGC CTA CTT CTT GGT CAT GAT CTT CAA GTA CTC-3′ from male and ovary cDNA, respectively. The reaction products were cloned into pUbi-Casper-SV40, which was created by inserting the SV-40 polyadenylation signal from pEGFP-N1 (Clontech, Palo Alto, California, United States) into pUbi-Casper2 (kind gift from Dr. Inge The). The vector for myc-tagged GFP expression (pKF63) was constructed similarly.

The vector for the expression of miR-277-responsive myc-YFP was constructed by first inserting the annealed oligonucleotides 5′-CAT GGA ACA AAA ACT TAT TTC TGA AGA AGA CTT GGG-3′ and 5′-CAT GCC CAA GTC TTC TTC AGA AAT AAG TTT TTG TTC-3′ into NcoI-cut pBSII-ITR1.1k-EYFP (a kind gift from Dr. Malcom Fraser) to add an N-terminal myc-tag. Then the vector was digested with NotI/XbaI and the annealed oligonucleotides 5′-GGC CTG TCG TAC CAG AGG ATG CAT TTA CAG TGT CGT ACC AGA GGA TGC ATT TAT GTC GTA CCA GAG GAT GCA TTT ACA GTG TCG TAC CAG AGG ATG CAT TTA-3′ and 5′-CTA GTA AAT GCA TCC TCT GGT ACG ACA CTG TAA ATG CAT CCT CTG GTA CGA CAT AAA TGC ATC CTC TGG TAC GAC ACT GTA AAT GCA TCC TCT GGT ACG ACA-3′ inserted, appending four miR-277 target sites to the 3′ UTR. Subsequently, the Pax6/EYFP/miR-277-target/SV-40-polyA cassette [68–70] was cloned into pPCar20.1 [71] creating pKF77. All of the described constructs were sequence verified. Transgenic flies were obtained by injection of pKF77 with Δ2–3 helper plasmid into *ry^506^* embryos using standard methods [72].

### S2 cell culture and RNAi

*Drosophila* S2 cells were the kind gift of Dr. Neal Silverman. The cells were cultured in Schneider's *Drosophila* medium (Life Technologies, Carlsbad, California, United States) supplemented with 10% FBS, penicillin-streptomycin mix (Life Technologies), and 0.2% of conditioned Schneider's medium. Transfection of plasmids was performed using siLentFect (Bio-Rad).

Gene fragments for the preparation of dsRNA were cloned into a Litmus28i vector (NEB) that was modified into a T/A cloning vector [73]. The following oligonucleotide pairs were used to obtain gene fragments: 5′-TTG GGC GAC GTT TTC GAG TCG ATC-3′ and 5′-TTT GGC CGC CGT GCA CTT GGC AAT-3′ for *dcr-1*; 5′-CTG CCC ATT TGC TCG ACA TCC CTC C-3′ and 5′-TTA CAG AGG TCA AAT CCA AGC TTG-3′ for *dcr-2*; 5′-ATG GAC CAG GAG AAT TTC CAC GGC-3′ and 5′-GGC CTC GTC GCT GGG CAA TAT TAC-3′ for *loqs*; 5′-ATA CAA TCT CCA CCA ATT TGT AGG-3′ and 5′-CGT CAA ATT ATT TAA AAT ATT TGT TTC-3′ for *r2d2*; 5′-AGC AGC AGC AGT GAT AGC GAT GGC-3′ and 5′-TCG GTT ATT TTA TTT GTT GCT TTA ATG-3′ for *Drosha*; 5′-GAT CAC ATG GTC CTG CTG GAG TTC GTG-3′ and 5′-CAG GTT CAG GGG GAG GTG TG-3′ for GFP. Gene fragments were amplified from the plasmid templates with both flanking T7 RNA polymerase promoters using oligonucleotides 5′-CTA TGA CCA TGA TTA CGC CAA GC-3′ and 5′-CAC GAC GTT GTA AAA CGA CGG CCA-3′. RNA synthesis from the PCR products was performed as described [74], and the phenol-extracted RNA products were denatured for 5 min at 95 °C and then re-annealed for 30 min at 65 °C. The concentration of dsRNA was estimated by native agarose gel electrophoresis and comparison to a DNA standard. S2 cells were seeded at 10^6^ cells/ml, and dsRNA was added directly to the growth medium at a final concentration of 10 μg/ml. After three days, additional dsRNA was added, and the cells were diluted 5-fold on the following day to permit further growth. Eight days after the initial dsRNA treatment, the cells were harvested by centrifugation, washed three times in phosphate-buffered saline, and re-suspended per ml of original culture in 15 μl of lysis buffer (30 mM HEPES-KOH [pH 7.4], 100 mM KOAc, 2 mM Mg(OAc)_2_), supplemented with protease inhibitors (Complete, Roche). The cells were disrupted either with 50 strokes of a Dounce homogenizer using a “B” pestle or by freeze/thawing. The extract was separated from debris by centrifugation at 18,000 × g for 30 min and aliquots frozen at −80 °C.

### In vitro pre-miRNA processing

Synthetic *Drosophila* pre-*let-7* bearing the characteristic end structure created by *Drosha* processing of pri-miRNA (5′-UGA GGU AGU AGG UUG UAU AGU AGU AAU UAC ACA UCA UAC UAU ACA AUG UGC UAG CUU UCU-3′) was 5′-^32^P radiolabeled with PNK, gel purified, and re-folded by heating at 95 °C for 2 min, then incubating at 37 °C for 1 h. Pre-*let-7* (20 nM) was incubated in a standard RNAi reaction with 50% (v/v) S2 cell lysate for 30 min. The reaction was deproteinized with Proteinase K and Phenol [74], then resolved by electrophoresis in a 15% denaturing polyacrylamide gel. Pre-*let-7* and *let-7* were quantified by phosphorimagery (BAS-5000; Fuji). The fraction processed (y) and time (t) was analyzed using Igor Pro 4.09A (Wavemetrics, Portland, Oregon, United States) by fitting the data to y = k_1_−k_1_e^(−k_2_^ ^t)^, where k_1_k_2_ corresponds to the initial velocity.

### Immunoprecipitation and immunoblotting

For immunoprecipitation, 100 μl of S2 cell extract were incubated with 2 μl affinity-purified antibody or 2 μl monoclonal anti-myc antibodies (clone 9E10, Sigma, St. Louis, Missouri, United States) for 30 min at 4 °C. Subsequently, protein A/G agarose (Calbiochem, San Diego, California, United States) or anti-rabbit IgG agarose (eBioscience, San Diego, California, United States) was added and the samples agitated at 4 °C for 90 min. For RNase treatment, RNase A was added to a final concentration of 50 μg/ml, and the samples incubated for 15 min at 4 °C prior to immunoprecipitation. Beads were washed four times with 1 ml of lysis buffer containing 1% (v/v) Triton X-100 (Sigma).

For Western blotting, the proteins were separated on 8% polyacrylamide/SDS gels and transferred to PVDF-membrane. All incubations and washes were in TBS containing 0.02% (v/v) Tween-20. For the rabbit primary antibodies, we used a secondary antibody that does not recognize the reduced form of rabbit IgG (Trueblot, eBioscience), permitting detection of Loqs, which migrates near the heavy antibody chain present in the immunoprecipitates.

To generate anti-Loqs antibody, two rabbits were immunized with the KLH-conjugated peptide MDQENFHGSSC. The specificity of the antibody was verified by Western blotting using extracts prepared from S2 cells transfected with the myc-Loqs PB expression vector, using untransfected S2 cell extract for comparison. Both rabbit anti-sera reacted with the over-expressed protein and against three small endogenous proteins. The antibody was affinity purified using the peptide antigen immobilized on agarose beads. Anti-Dcr-2 antibody was raised in chicken using the KLH-conjugated peptide CNKADKSKDRTYKTE. IgY was affinity-purified from egg yolk using peptide antigen immobilized on agarose beads. Anti-Drosha antibody was kindly provided by Greg Hannon [10].

### Gel-filtration chromatography

200 μl of S2 cell extract was separated by chromatography on a Superdex-200 HR 10/300 GL column (Amersham-Pharmacia) using a BioCad Sprint (PerSeptive Biosystems, Framingham, Massachusetts, United States) as described [75]. Protein from three-quarters of every other fraction was precipitated with 10% (v/v) trichloroacetic acid and 0.001% (w/v) deoxycholate and analyzed by Western blotting. The remainder of each fraction was analyzed for pre-miRNA processing activity.

### Analysis of YFP reporter fluorescence and eye color using the white-IR transgene

Fluorescence and normal light images were taken with a Leica MZ-FLIII stereomicroscope equipped with a cooled color CCD-camera (Firecam, Leica, Wetzlar, Germany). The control animals expressing YFP without the miR-277 target sites contained a pBAC3xP3-EYFP, p-Gal4Δ-K10 insertion on the X chromosome [66]. Maximal pixel intensity was determined using ImageGuage 4.2 (Fuji). The average intrinsic background fluorescence present in Oregon R eyes (*n* = 4) was subtracted from the value determined for each YFP-expressing eye. Eye pigment was measured as described [76]. The heads of 10 males (3–4 d post eclosion) of each genotype were manually dissected. For each genotype, five samples of two heads each were homogenized in 0.1 ml of 0.01 M HCl in ethanol. The homogenates were placed at 4 °C overnight, warmed to 50 °C for 5 min, clarified by centrifugation, and the optical density at 480 nm of the supernatant measured relative to the value for the Oregon R stock.

### Analysis of stellate expression in testes and determination of hatch rates and immunofluorescence microscopy

Stellate expression and hatch rates were analyzed as described previously [24]. Immunofluorescence microscopy was as described previously [77]. Spectrosome and fusome were labeled with monoclonal antibody 1B1 (Developmental Studies Hybridoma Bank, Iowa City, Iowa, United States), as described by Lin and Spradling [78].

## Supporting Information

Figure S1 A Concentration Series Generated by Dilution of the Eye Pigment Extract from Oregon R Flies The concentration of each sample, relative to the undiluted sample, was plotted versus its absorbance at 480 nm. The data were fit to a line using Igor Pro 5.01.
(622 KB EPS).

Figure S2 Loqs Disrupts Germ-Line Stem Cell Maintenance Wild type and *loqs* mutants were labeled for α-Spectrin and Actin. In the merged images, α-Spectrin is green; Actin is red. Two examples of wild-type and *loqs* ^f00791^ mutant germaria are shown. In wild type, anti-α-Spectrin labeled both the spectrosome, a spherical structure unique to the germ-line stem cells and their daughters, the cystoblasts, and the highly branched fusome found in the cystocytes. The stem cells are located at the anterior of the germarium. In germaria isolated from 3 to 4 day-old *loqs* mutant females, none of the cells showed a prominent spectrosome, although fusome was detected. Thus, stem cells were originally present but were not maintained. The germ-line cells that remain appear to be cystocytes. The muscle sheath surrounding the ovarioles stains intensely for Actin. α-Spectrin was labeled with a monoclonal antibody; filamentous Actin was labeled with rhodamine-phalloidin.
Scale bar in the upper right panel = 10 μm.
(4.8 MB EPS).

### Accession Numbers

The Arabidopsis Information Resource (http://www.arabidopsis.org) accession numbers for the genes and gene products discussed in this paper are: DCL1 (AT1G01040) and Hyl1 (AT1G09700).

The Ensembl (http://www.ensembl.org/Homo_sapiens) accession numbers for the genes and gene products discussed in this paper are: *C. elegans rde4* (T20G5.11) and *dcr1* (K12H4.8), human DGCR8 (NSG00000128191), Ago2 (ENSG00000123908), Exportin5 (ENSG00000124571) and TRBP (ENSG00000139546), and mouse PRBP (ENSMUSG00000023051).

The FlyBase (http://flybase.bio.indiana.edu) accession numbers for the genes and gene products discussed in this paper are: *ago1* (CG6671, FBgn0026611), *ago2* (CG7439, FBgn0046812), *armi* (CG11513, FBgn0041164), *aub* (CG6137, FBgn0000146), *dcr-1* (CG4792, FBgn0039016), *dcr-2* (CG6493, FBgn0034246), *drosha* (G8730, FBgn0031051), *loqs* (CG6866, FBgn0032515), *pasha* (CG1800, FBgn0039861), *piwi* (CG6122, FBgn0004872), *r2d2* (CG7138, FBgn0031951), *spnE* (CG3158, FBgn0003483), *Stellate* (FBgn0003523), *Su(Ste)* (FBgn0003582), *vasa* (CG3506, FBgn0003970), and *white* (CG2759, FBgn0003996).

The Rfam (http://www.sanger.ac.uk/Software/Rfam/mirna/index.shtml) accession numbers for the genes and gene products discussed in this paper are: *bantam* (MI0000387), *let-7* (MI0000416), miR-277 (MI0000360), miR-7 (MI0000127), and TAR (RF00250).

## Abbreviations

dsRBD: double-stranded RNA binding domain
dsRNA: double-stranded RNA
GFP: green fluorescent protein
IR: inverted repeat
miRNA: microRNA
PA: protein isoform A
PB: protein isoform B
PC: protein isoform C
pre-miRNA: precursor miRNA
pri-miRNA: primary miRNA
RA: RNA splice variant A
RB: RNA splice variant B
RC: RNA splice variant C
RISC: RNA-induced silencing complex
RNAi: RNA interference
S2: Schneider-2
siRNA: small interfering RNA
TAR: trans-activator RNA
YFP: yellow fluorescent protein
